# Appropriateness of packed red blood cells transfusions in chronic anemic patients in the emergency department: the TRANSFUS-ED retrospective analysis

**DOI:** 10.1007/s11739-023-03277-w

**Published:** 2023-04-22

**Authors:** Matteo Paganini, Fabio Rigon, Federico Rebustello, Vito Cianci, Irene Bertozzi, Maria Luigia Randi

**Affiliations:** 1 Department of Biomedical Sciences, University of Padova, Via Marzolo, 3, 35131 Padua, Italy; 2 Medical School, University of Padova, Via Giustiniani, 2, 35128 Padua, Italy; 3 Emergency Department, Azienda Ospedaliera di Padova, Via Giustiniani 2, 35128 Padua, Italy; 4 First Clinical Medicine, Department of Medicine (DIMED), Via Giustiniani, 2, 35128 Padua, Italy

**Keywords:** Emergency medicine, Anemia, Blood transfusion, Cost analysis

## Abstract

Patients suffering from chronic anemia can benefit from scheduled transfusions of packed red blood cells (PRBCs), while urgent transfusions have specific indications. These patients frequently seek medical attention in the emergency department (ED), where they can be inappropriately transfused, but research in this field is limited. This study aimed to assess the appropriateness of PRBCs transfusions in chronic anemic patients in the ED. A retrospective analysis was performed on patients who accessed the ED of the Azienda Ospedaliera di Padova (Padova, Italy) between 2016 and 2019 and received PRBCs transfusions. Patients aged ≥ 18 years old and with chronic anemia were included, while those with acute anemia or admitted to the hospital after the transfusion were excluded. Chronic anemia was defined as satisfying one of the following in the past medical history: diagnosis of chronic anemia; two or more previous blood samplings demonstrating anemia; periodic transfusions. As primary outcome, the appropriateness of transfusions was assessed according to the American Association of Blood Banks (AABB) 2016 criteria, using the recommended threshold of 7 g/dL for hemodynamically stable adults and 8 g/dL for patients with pre-existing cardiovascular disease. Out of 1153 transfusions, 344 transfusions were included in the study. According to our criteria, 139 (40.4%) patients were inappropriately transfused, resulting in a total estimated cost of 54,528.71 € in the study period. This study showed that transfusions in chronic anemic patients are recurrent events in the ED and are frequently inappropriate. A possible explanation could be the lack of a well-structured primary care network granting periodic transfusions in ambulatory centers. In the future, implementing and improving chronic anemic patients’ access to transfusion services through dedicated pathways could reduce the burden on the ED and also decrease costs.

## Background

Anemia is one of the most common diseases, affecting about one-quarter of the global population [1]. Preschool-age children (1–4 years) and women are the most affected groups all over the world [1], but up to 17% of people over 65 years are estimated to suffer from anemia [2]. Despite such a high prevalence, anemia is an underrecognized public health issue with a significant clinical impact [3].

Acute anemia developing from hemorrhage requires emergent transfusion of packed red blood cells (PRBCs) and bleeding source control. Conversely, chronic anemia usually requires a thorough differential followed by a multimodal treatment, especially in the elderly [4].

Subjects with chronic anemia can benefit from periodic transfusions in ambulatory centers, scheduled after regular hemoglobin (Hb) checks. Current guidelines issued by the American Association of Blood Banks suggest PRBCs transfusion in stable and asymptomatic patients with a Hb of 7 g/dL or less (restrictive strategy). A threshold of 8 g/dL is reserved for orthopedic or cardiac surgery patients and those with pre-existing cardiovascular disease [5]. Conversely, transfusions for chronic anemic patients with symptoms such as fatigue, dyspnea, orthostatic hypotension, or tachycardia and a Hb < 10 g/dL is generally not indicated but should be considered after clinical evaluation [5].

Emergency Departments (EDs) account for 10.5% [6]—29% [7] of total hospital transfusions, most of the time due to urgent conditions such as bleeding. However, transfusions can be inappropriate in many cases due to wrong indications or excessive number of PRBCs administered [8]. Among these, chronic anemic patients represent a non-negligible group [9], especially if asymptomatic, but their actual contribution to overall costs and patient processing delays in the ED is still unclear.

Thus, this study aimed to assess the appropriateness of PRBCs transfusions in chronic anemic patients in the ED of an academic, tertiary care hospital in Northeast Italy. Further evaluations included the cost analysis and the differences between the appropriately and inappropriately transfused patients in terms of presence of symptoms and type of presentation.

## Methods

This is a retrospective analysis conducted in the ED of the Azienda Ospedaliera Università di Padova (Padova, Veneto Region, Italy). This ED has approximately 120,000 visits/year, is run by internist and emergency medicine physicians, and is part of a hub hospital and trauma center with 1450 inpatient beds.

### Study population

**Fig. 1:**
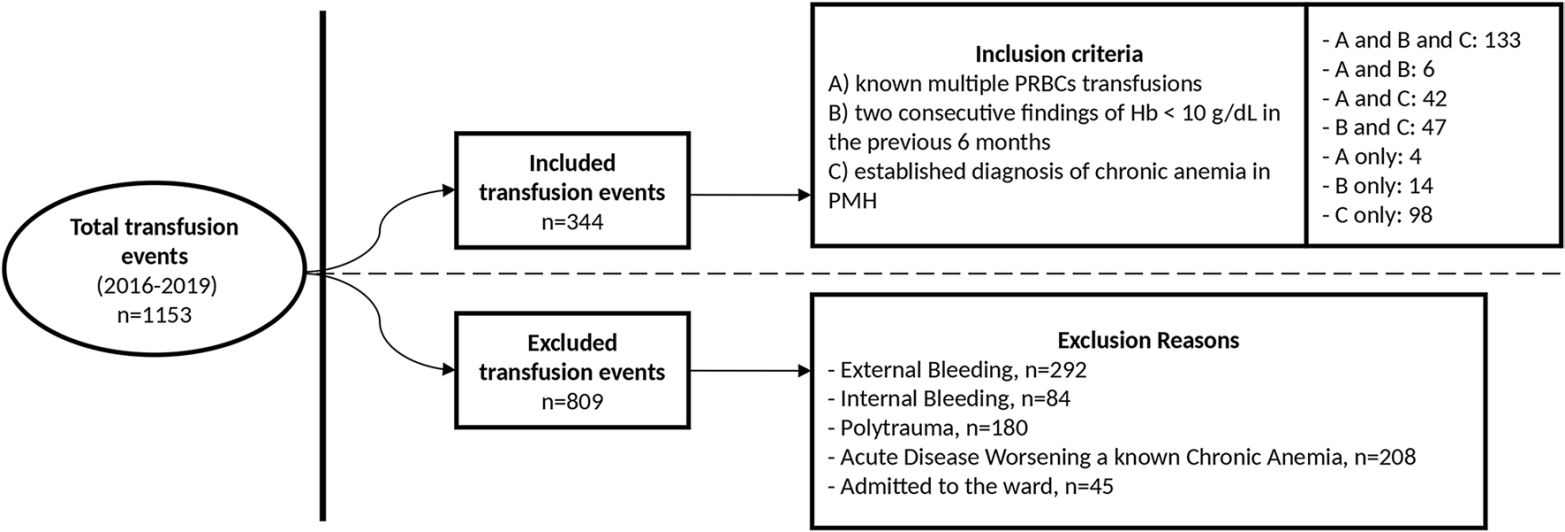
Patients’ inclusion and exclusion flowchart

All the patients that visited the ED and received a PRBC transfusion between January 1st, 2016 and December 31st, 2019 were considered eligible, and included if meeting the following criteria:

• Age ≥ 18 years;
• Suffering from chronic anemia as defined by any one of the following conditions: known multiple PRBCs transfusions; two consecutive findings of Hb < 10 g/dL in the previous 6 months; or established diagnosis of chronic anemia in the past medical history (Fig. 1).

Patients were instead excluded if presenting with: external (e.g., hematemesis, melena, epistaxis) or internal bleeding (e.g., hemothorax, hemoperitoneum, hematoma); polytrauma; an acute disease worsening their chronic anemia (e.g., acute renal impairment, sepsis, hematologic disease). Patients admitted to the wards were also excluded (Fig. 1).

### Data collection

Data were manually extracted by the investigators from individual patient charts and encoded to a master sheet using a Microsoft Office Excel spreadsheet (Version 2016, Microsoft Corporation, Redmond, WA). Demographics and past medical history—focused on diseases predisposing to chronic anemia and cardiovascular diseases, and multiple presentations to the ED—were derived from each patient’s digital chart. Complete blood count, symptoms, day of the week of presentation, arrival modality, number of PRBCs infused, immediate adverse reactions to transfusions, and time elapsed from medical evaluation to discharge (length of stay) were extracted from the report of each ED presentation. Anemia severity at presentation was described according to the World Health Organization’s criteria as mild (Hb ≥ 11 g/dL), moderate (8 ≤ Hb < 11 g/dL), or severe (Hb < 8 g/dL) [10].

Patients were then stratified according to the appropriateness of transfusion (appropriate or inappropriate) using the American Association of Blood Banks (AABB) 2016 guidelines [5]. Specifically, transfusions administered to chronic anemic patients were classified as inappropriate if having the following characteristics:

- Asymptomatic patients without known cardiovascular ischemic disease (e.g., previous acute coronary syndrome, coronary artery bypass graft, percutaneous coronary intervention) and a Hb > 7 g/dL and ≤ 8 g/dL;
- Asymptomatic patients with Hb > 8 g/dL and < 10 g/dL;
- Patients with Hb ≥ 10 g/dL.

Of note, fatigue alone (e.g. without exertion dyspnea, chest pain, dizziness) was tracked as variable but not considered as a major symptom.

### Ethical considerations

The study was granted exemption from requiring ethics approval by the jurisdictional ethics committee of the “Azienda Ospedaliera di Padova” in Padova, Italy (No. 73989/21). All data were collected such that individual subjects could not be identified or exposed to risks or liabilities. The study was conducted aiming to reduce carbon footprint and increase research sustainability by reducing travel, using virtual meeting tools, and consulting digital charts without printing them. The datasets generated and analyzed during the current study are available from the corresponding author upon request.

### Data analysis

Quantitative data were tested for normality with a Shapiro–Wilk test and presented as median and interquartile range (IQR). Qualitative data were analyzed descriptively through their distribution frequency and compared using a chi-squared test where appropriate. *P* values < 0.05 were considered statistically significant. Statistical software JASP (ver 0.11.1; JASP Team, 2019) was used to analyze the results.

Costs of inappropriately transfused PRBCs are reported in Euro (€) and were calculated using the institution blood bank’s reported annual per-unit costs (200 €) multiplied by the number of units. The estimated cost of the ED admission was calculated through hospital administration. Considering the personnel involved (hourly wage for one doctor and one nurse), the materials used (e.g., tubes for blood samples, venous peripheral access), and the exams/procedures performed (e.g., complete blood count, renal function, blood typing, telemonitoring, patient counseling), each admission accounted for 95.89 €.

## Results

In the study period, 1153 transfusion events in the ED were selected as eligible, and 344 (29.8%) transfusions in chronic anemic patients were included (Fig. 1).

Specifically, 288 patients accessed the ED and received a PRBC transfusion; of them, 41 re-accessed the ED for the same reason during the study period, with a median time of 126 days (IQR: 50–267 days) between each re-access. Median age was high (81 years old) and most of the patients were female; 20.5% suffered from a hematological disease causing their chronic anemia, and 63% had a cardiovascular comorbidity (Table 1).

**Table 1:** Baseline characteristics of chronic anemic patients transfused in the emergency department (ED)

Type of presentation to the ED (number of patients)	
Single presentation	247
≥ 2 presentations	41
Age (years)	81 (72–88)
Gender (Female), *n* (%)	159 (55.2)
Referral from nursing homes, *n* (%)	38 (13.2)
Underlying disease causing chronic anemia, *n* (%)	
Hematological	59 (20.5)
Oncological	56 (19.4)
Chronic renal failure	37 (12.8)
Chronic inflammatory disease	33 (11.5)
Not specified	131 (45.5)
Cardiovascular disease, *n* (%)	63 (21.9)

Of the 288 patients included, 41 presented multiple times, totaling 344 transfusion events included in the study

Among the included cases, none had mild anemia (Hb ≥ 11 g/dL) [10]. Most of the included patients were symptomatic at presentation, and fatigue was the most prevalent complaint (Table 2). Of note, the median length of stay for the included events was 440 minutes (7.5 h) but reached a maximum of 1300 minutes (about 21 h).

**Table 2:** Baseline data of the 344 transfusion events in the emergency department (ED)

Severity of chronic anemia, *n* (% of total cases) [10]	
Moderate (8 ≤ Hb < 11 g/dL)	119 (34.6)
Severe (Hb < 8 g/dL)	225 (65.4)
At least one symptom, *n* (% of total cases)	212 (61.6)
Symptomatic cases per severity of chronic anemia, *n* (% on severity class)	
Moderate (8 ≤ Hb < 11 g/dL)	72 (60.5)
Severe (Hb < 8 g/dL)	140 (62.2)
Symptoms, *n* (% total cases)	
Fatigue	162 (76.4)
Dyspnea	67 (31.6)
Chest pain	19 (8.9)
Dizziness	15 (7.1)
Palpitations	10 (4.7)
Headache	4 (1.9)
Syncope	2 (0.9)
ED presentation modality, *n* (% total cases)	
Physician referral	201 (58.4)
Patient decision	143 (41.6)
Length of stay in the ED, minutes (range min–MAX)	440 (378–521)
PRBCs units transfused, *n* (units/patient)	576 (1.67)

Most of the included patients suffered from severe chronic anemia (65.4%). A total of 576 packed red blood cells (PRBCs) were transfused

About half of the patients (58.4%) were referred to the ED by a physician (general practitioner or another specialist) (Table 2). After stratifying symptoms at presentation for this variable, a statistically significant correlation was found between spontaneous presentation to the ED and complaining of dyspnea, chest pain, or dizziness. Instead, fatigue as an isolated symptom was significantly more reported in patients referred by a physician (Table 3).

**Table 3:** Symptoms stratified by modality of presentation to the emergency department (ED)

Symptoms at presentation, *n* (%)	Modality of presentation		*p*
	Physician referral (*n* = 201)	Patient decision (*n* = 143)	
At least 1 symptom registered	107 (53.2)	105 (73.4)	*p* < 0.001
Fatigue and any other symptom	95 (47.3)	67 (46.9)	n.s
Fatigue as an isolated symptom	73 (36.3)	37 (25.9)	*p* = 0.041
Dyspnea	23 (11.4)	44 (30.8)	*p* < 0.001
Chest pain	2 (1.0)	17 (11.9)	*p* < 0.001
Dizziness	3 (1.5)	12 (8.4)	*p* < 0.001
Palpitations	6 (3.0)	4 (2.8)	n.s
Headache	2 (1.0)	0 (1.4)	n.s
Syncope	0 (0.0)	2 (1.4)	n.s

According to the criteria, 59.6% of the transfusions (205) were appropriately administered, mostly to patients with a Hb of less than 7 g/dL. Conversely, all the 139 inappropriate transfusions (except 1) happened at Hb levels between 7 and 10 g/dL, in asymptomatic patients (Table 4).

**Table 4:** Stratification of the included 344 transfusions according to the appropriateness criteria

Inappropriate transfusions, *n* (% of the total events)	139 (40.4)
Asymptomatic patients with Hb > 7 and ≤ 8 g/dL and without known cardiovascular ischemic disease, *n* (%)	70 (50.4)
Asymptomatic patients with Hb > 8 and < 10 g/dL, *n* (%)	68 (48.9)
Patient with Hb ≥ 10 g/dL, *n* (%)	1 (0.7)
Appropriate transfusions, *n* (% of the total events)	205 (59.6)
Patients with Hb ≤ 7 g/dL, *n* (%)	120 (58.5)
Symptomatic patients with Hb > 7 and ≤ 8 g/dL, *n* (%)*	47 (23.0)
Symptomatic patients with Hb > 8 and ≤ 10 g/dL, *n* (%)	38 (18.5)

*Hb:* hemoglobin

*Including 28 affected by ischemic cardiac disease

No significant association was noted between comorbidities and the appropriateness of transfusions, except for cardiovascular disease—which was more prevalent in the appropriate transfusions group (Table 5).

**Table 5:** Cases stratified by transfusion appropriateness in the emergency department (ED)

	Appropriate (*n* = 205)	Inappropriate (*n* = 139)	*p*
Underlying disease causing chronic anemia, *n* (%)﻿			
Hematological	53 (25.8)	28 (20.1)	n.s
Oncological	33 (16.1)	30 (21.6)	n.s
Chronic renal failure	32 (15.6)	19 (13.7)	n.s
Chronic inflammatory disease	21 (10.2)	17 (12.2)	n.s
Cardiovascular disease, *n* (%)	58 (28.3)	19 (13.7)	*p* < 0.001
ED presentation modality, *n*. (% total cases)			
Physician referral	106 (51.7)	95 (68.3)	*p* = 0.002
Patient decision	99 (48.3)	44 (31.7)	
Length of stay in the ED, minutes (range min–MAX)	442 (381–521)	433 (374–519)	n.s

Cardiovascular disease was associated with appropriate transfusions

Regarding the symptoms and appropriateness, no significant associations were found between symptoms at presentation and appropriateness, except for fatigue as an isolated symptom and inappropriate transfusions (*p* = 0.044).

### Cost analysis

During the observation period, 206 PRBC units are estimated to have been inappropriately transfused, equaling a cost of 41,200 €. The associated costs for personnel, materials, and exams/procedures consumables accounted for 13,328.71 €, totaling 54,528.71 € for the whole study period.

## Discussion

Chronic anemic patients require periodical follow-up and planned transfusions to maintain a good quality of life. Most can be transfused in dedicated ambulatory centers after establishing specific agreements between primary care, specialists, and local blood banks. Instead, in case of acute severe symptoms, urgent transfusions are usually warranted after being evaluated by an emergency medicine physician that can opt for transfusion in the ED, admission to the ward, or timely referral to the transfusion clinic. Another recently studied option is intravenous iron administration in iron deficiency anemia in the ED [8], but also oral supplementation can be prescribed to prevent exacerbations and reduce PRBCs transfusions. A considerable amount of literature has been published on transfusions in emergency settings, especially in trauma patients and hemorrhagic shock. On the other hand, PRBC transfusion in chronic anemia is currently a neglected topic, especially in the ED, where this subset of patients frequently seek medical attention [8, 9].

This is the first study to specifically assess the appropriateness of PRBC transfusions in chronic anemic patients in a large ED in Northern Italy. Several factors could have contributed to the high proportion of inappropriate PRBC transfusions (40.4% of cases) found in the investigated ED.

First, guidelines at both national and international levels pose specific indications in case of emergent or urgent situations but are unclear regarding chronic anemic patients [5, 11]. Symptoms triggering PBRCs transfusions are still not detailed but only described as the manifestation of inadequate oxygen delivery, often leaving physicians in the middle of clinical decision dilemmas. Moreover, current transfusion thresholds are mostly guided by studies performed on inpatients. In this study, inappropriate transfusions mostly happened in patients without major symptoms and presenting with a Hb between 7 and 10 g/dL (Table 4). Interestingly, fatigue as an isolated complaint was significantly associated with the ED referral by a primary care physician (Table 3) and transfusion inappropriateness. Among possible explanations, fatigue could have been misinterpreted as a major symptom of a severe underlying condition, prompting patients' inappropriate referral to the ED and transfusion. In addition, the failure of the local outpatient follow-up network or patients' loss of trust in primary care [15] could have increased ED referrals, spontaneous presentations, or patients' requests for transfusions in the ED.

It is known that the older population experiences a decline in out-of-home mobility [12] and has a greater prevalence of hematologic or neoplastic diseases, predisposing them to a worsening chronic anemia [13, 14]. Thus, the high median age of the included sample (81 years; IQR: 72–88 years) could have induced clinicians to inappropriately transfuse patients even without severe symptoms, despite Hb values above 7 g/dL, or maybe to meet patients’ needs and improve their satisfaction.

Inappropriate transfusions' costs are underestimated by the study’s retrospective nature but accounted for 41,200 € for PRBCs alone and a total cost of 54,528.71 €. This is particularly important in universal national health systems, where cost-effectiveness impacts the budget and availability of treatments to patients.

Of note, the mean length-of-stay of transfusion events was 7.5 h, potentially affecting patient processing. In fact, from an ED management perspective, transfusions are complex and time-consuming procedures entailing nurse and medical evaluation, time to obtain blood samples, results, and blood crossmatching, time to receive and transfuse PRBCs, and time to re-check Hb levels reached. Although it was not possible to retrospectively investigate patient processing outcomes, it is reasonable to think that over-conservative referrals to the ED and inappropriate transfusions impacted patient throughput and contributed to a public health problem called ED overcrowding [16, 17]. Further in-depth analysis could provide solutions to mitigate ED overcrowding due to inappropriate transfusions, such as implementing "fast-track anemia clinics" [9, 18–20] to divert chronic anemic patients not needing urgent transfusions from the ED and preserving its functional resilience.

This study has several limitations. The retrospective nature hampered further investigations regarding the factors affecting transfusion appropriateness among emergency medicine physicians, referrals, and recurring ED presentations. Such results may have been influenced by factors not reported in charts, such as relevant symptoms, logistic constraints, or patient preference. Patient satisfaction after transfusions was not tracked as well. Given the relatively small number of patients included and the monocentric approach, results must be interpreted carefully, and the conclusions may not apply to other EDs. However, this is the first study to specifically assess such an underestimated problem in an Italian ED, and future investigations will verify these preliminary findings.

## Conclusion

This study is the first to investigate and find a high proportion of inappropriate PRBCs transfusions in chronic anemic patients in a Northern-Italian ED. Despite the limited value of a monocentric and retrospective study, these findings could help start quality improvement projects, aiming to educate chronic anemic patients, GPs, and emergency medicine physicians on wiser use of PRBCs, reducing costs and the burden on the ED.

## Data Availability

The datasets generated during and/or analyzed during the current study are available from the authors upon reasonable request.
